# Evaluation on the Corrosion of the Three Ni-Cr Alloys with Different Composition

**DOI:** 10.1155/2011/397029

**Published:** 2011-03-03

**Authors:** Srinivasa B. Rao, Ramesh Chowdhary

**Affiliations:** ^1^Department of Prosthodontics, Mamata Dental College and Hospital, Khammam 507002, India; ^2^Department of Maxillofacial Prosthodontics, S. Nijalingappa Institute of Dental Sciences & Research Centre, Gulbarga 585105, India

## Abstract

Dental casting alloys are widely used in contact with oral tissue for many years now. With the development of new dental alloys over the past 15 years, many questions remain unanswered about their biologic safety. Concepts and current issues concerning the response to the biologic effects of dental casting alloys are presented. In this paper, samples of three commercially available nickel-chrome (Ni-cr) casting alloys (Dentaurum, Bego, Sankin) were taken to assess their corrosion behavior, using potentiodynamic polarization method (electrochemical method) with fusayama artificial saliva as an electrolyte medium to check for their biocompatibility. The parameters for corrosion rate and corrosion resistance were obtained from computer-controlled corrosion schematic instrument, namely, potentiostat through corrosion software (power CV). The results obtained were analyzed by classic Tafel analysis. Statistical analysis was done by Student's *t*-test and ANOVA test. It was concluded that Dentarum and Bego showed satisfactory corrosive behavior, with exception of Sankin which depicted higher corrosion rate and least resistance to corrosion. Thus, the selection of an alloy should be made on the basis of corrosion resistance and biologic data from dental manufactures.

## 1. Introduction

Dental casting alloys are widely used in dentistry, especially in contact with oral tissues for many years. It is of paramount importance to understand and get familiar with biocompatibility of casting alloys for their long-term success in rendering successful treatment for the patients. One of the most relevant properties of a casting alloy to its biologic safety is its corrosion resistance [1]. Research in this area has generated as many questions as it has answered, and much more needs to be known about the biocompatibility of these alloys.

In view of the extensive use of base metal alloys as an alternative to conventional gold-based alloys [2], an effort should be made to evaluate the corrosion properties. This ultimately answers the question of performance for a longer period of time in oral cavity. Alloys, such as copper-aluminium [3], copper-silver [4], and copper-Zinc [5], were subsequently rejected because of their higher corrosive property. Nickel-chrome (Ni-cr) casting alloys were developed as an alternate, because of their superior properties in relation to porcelain-fused-to-metal (PFM) applications, high strength for crowns and fixed partial denture, better elongation percentage, and high elastic modulus for removable partial denture [6]. Electrochemical studies have shown that Ni-cr casting alloys do corrode in physiological solution such as balanced salt, protenacious solution, artificial saliva, and human saliva [7]. Contrary to this, it is also reported that there is good corrosion resistance for Ni-cr casting alloys in the oral cavity [8]. It has been suggested that 16%–27% chromium provides an adequate protective oxide film for these nickel-based alloys. Alloys with lower chromium content may not be able to adequately develop oxide films for corrosion resistance in the oral milieu [9].

To evaluate the corrosion behavior of biomaterials, various in vitro methodologies have been used quantitively to check for their biocompatibility. Electrolytes like fusayama's artificial saliva, 1% Nacl, darvells solution, cell culture medium, and so forth [10], with methodologies like electrochemical techniques and potentiodynamic polarization method, are used to evaluate the corrosion rate and corrosion resistance [11]. Surface analysis (X-ray photoelectron spectroscopy) [12] and other methods like cytotoxicity and analysis of elements released into the solution [13], atomic absorption spectrophotometry [14], and zonal coulometric analysis [15] have also been used.

Potentiodynamic polarization method, chosen for this study, is an acceleration test, which helps manufacturers screen dental casting alloys more rapidly, and its theory provides new formulations to the clinician, more expediently [16]. In this study, it was planned to evaluate the corrosive behavior of three different commercially available Ni-cr casting alloys, by using potentiodynamic polarization method.

## 2. Materials and Methods

An in vitro study was carried out for the evaluation of corrosive behavior, that is, corrosion rate and corrosion resistance of different commercial manufacturers of Ni-cr casting alloys, using potentiodynamic polarization method. The Ni-cr alloys selected for this study were from Wiron 99 (Bego, Bremen, Germany), Remanium cs, (Dentaurum, Springen, Germany), and CB Soft (Sankin, Dentsply, York, USA) (Table 1).

The samples of commercial Ni-cr alloys of 10.0 × 2.0 × 1.0 mm dimensions in size were polished incrementally from 400 grit emery paper to 1000 grit emery paper and then washed with acetone and distilled water in an ultrasonic cleaner. Prior to the experiments, samples were placed in airtight plastic bottles to prevent contamination.

Fusayama artificial saliva was used as an electrolyte medium (Table 2). Fusayama artificial saliva solution constituents closely resemble those of natural saliva. During the study, the artificial saliva solution temperature was maintained at room temperature of 25°C [16].

Five samples, each of 3 different companies' alloys, were made (Remanium CS (Dentaurum), Wiron 99 (Bego), and CB Soft (Sankin)) in total 15 in number. All samples were individually soldered to copper wire to pass an electric current with a Tygon tubing which is attached to glass tube. Ni-cr casting alloy test sample was selected as working electrode, platinum as a standard electrode, and saturated calomel electrode as reference electrode. These sample electrodes were placed in a cell with a few millimeters (mm) apart in artificial saliva (fusayama) as an electrolyte medium. All the electrodes: the working electrode—Ni-cr casting alloys, and reference electrode standard electrode—platinum, saturated calomel electrode of 1 square centimeter (sqcm), were dipped in electrolyte medium—artificial saliva.

### 2.1. Potentiodynamic Scan

Potentiodynamic scan is designed for electrochemical applications that require relatively large current and high-compliance voltage such as battery studies, corrosion, electrolysis, and electroplating. The power range is ±2 A, and the compliance voltage is ±25 V. The potential of a corroding metal, often termed *E*_corr_, is probably the single most useful variable measured in corrosion studies as well as during the corrosion monitoring of complex field situations. It is readily measured by determining the voltage difference between a metal immersed in a given environment and an appropriate reference electrode. The scan was performed at the corrosion potential (*E*_corr_). That is, the rate of oxidation exactly equals to the rate of reduction. Scanning is performed at rate of 1 mv/second up to +1 volt (anodic polarization), to determine “a” as anodic Tafel slope constant, and up to −1 volt (cathodic polarization), to determine “c” as cathodic Tafel slope constant, and it then reverses back to *E*_corr_ corrosion potential. The result of particular experiment processed by microcomputer is displayed as polarization curves. The resultant graphs were analyzed using classic “Tafel analysis” which is displayed as log of current on *X*-axis and potential on *Y*-axis.

### 2.2. Classic Tafel Analysis

Classic Tafel analysis helps in the interpretation of polarization curves and is performed by extrapolating the linear portions of a log current versus potential to their intersection at an anodic and cathodic current that determines the *I*_corr_

(1) $$\text{Corrosion}\;\text{rate}\;\left(\text{mpy}\right)=0.13\;I_{\text{corr}}\;\left(\text{EW}\right)\;\text{K}/\text{d},$$

where EW = 22.4 is equivalent weight of corroding specimen, d = 8 is density of corroding specimen, *I*_corr_ is corrosion current density A/cm^2^, and K is corrosion constant 1.288 × 10^5^.

## 3. Results

Quantitative values of corrosion rate (mpy) were ranked and compared using Student's *t*-test and ANOVA test. The values for corrosion rate were recorded (Table 3).

According to test sequence, which is obtained from *E*_corr_ (corrosion current potential) values (Table 4), *I*_corr_ (corrosion current density) values (Table 5) were measured, using classic Tafel analysis. Even values for corrosion resistance (K cm^−2^) were recorded (Table 6).

**Figure 1:**
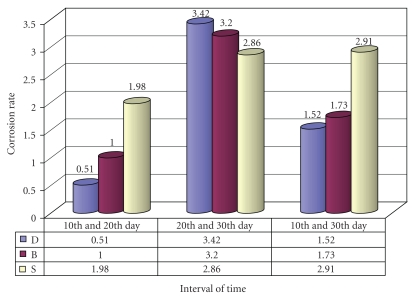
Comparison of corrosion rate (*t*-values) of casting alloys at various intervals of time.

Statistical analysis for corrosion rate of different company samples, namely Remanium CS (Dentaurum), Wiron 99 (Bego), and CB Soft (Sankin) were analyzed at various intervals of time using Student's *t*-test (Figure 1). For Remanium CS (Dentaurum), the “*t*” calculated values between the 10th and 20th, 20th and 30th, and 10th and 30th days are 0.51, 3.42*, and 1.52, respectively. For Wiron 99 (Bego) and the “*t*” calculated values between the 10th and 20th, 20th and 30th, and 10th and 30th days are 0.59, 3.20*, and 1.73, respectively. Finally for CB Soft (Sankin), the “*t*” calculated values between the 10th and 20th, 20th and 30th, and 10th and 30th days are 1.98, 2.86*, and 2.91, respectively.

**Figure 2:**
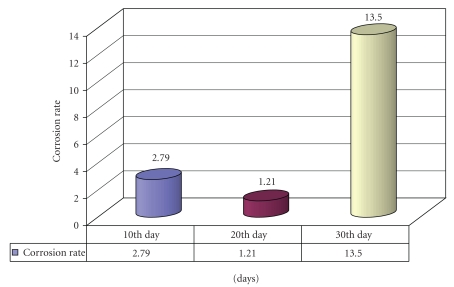
Comparison of corrosion rate of nickel-chrome casting alloys at various intervals of time.

Statistical analysis of Ni-cr casting alloys at various intervals of time is calculated by using ANOVA test and is tabulated. By the assessment of corrosion rate (mpy), the calculated “*f*” values on the 10th, 20th, and 30th days are 2.79, 1.21, and 13.5*, respectively (Figure 2).

## 4. Discussion

Corrosion of dental alloys is a complex process, depending not only on alloy's composition and structure, but also on many other factors such as surface treatment, environmental conditions around the alloy, and composition of surrounding electrolyte selected for the study [1, 17–20].

However, for specific environment, corrosion depends on the structure and composition of the alloy [1, 21]. The structure of the alloy, whether in single or multiple phases, is an important factor for its corrosion rate [1]. On the other hand, some alloying elements are very prone to enhance the behavior of corrosion, resulting in the release of elements into the electrolytes and thus increasing or decreasing the corrosion rate [1, 22–24]. Compromising these physical properties leads to an increase in biological irritation.

Remanium CS resulted in lower corrosive rate. This can be explained by its higher percentage of chromium. Chromium as chromium oxide (Cr_2_O_3_) and molybdenum as molybdenum oxide (Mo_3_) provide the initial stability to prevent dissolution of metal ions and thus provide resistance to corrosion and lesser corrosive rate. Wiron 99 was the next best among the Ni-cr casting alloys. This can be explained by its low percentage of chromium 22.5 wt% and molybdenum 9.5 wt% compared to Dentaurum's Remanium CS. Chromium as (Cr_2_O_3_) and molybdenum as (MO_3_) help in the formation of stable surface oxide film. Sankin's CB Soft showed the highest corrosion rate amongst all the samples selected for the study. There is a huge variation in its chemical composition when compared to the rest of the alloy samples. Less amount of chromium content, that is, 4.9 wt% and the absence of molybdenum element in CB Soft (Sankin) resulted in the absence of surface oxide passive film formation onto the metal surface.

Thus, the composition and integrity of the surface oxide film on Ni-cr casting alloy are critical for their performance as dental restoration. The results showed that Ni-cr casting alloys with a higher chromium and molybdenum content have much higher passive range and are immune to corrosion. As demonstrated by the results of Al-Hiyasat et al., Remanium CS had the least cytotoxicity and CB Soft the most [18]. This depends not only on the chemical composition but also on the characterization of passive film on the alloys [14].

A study conducted by Leung and Darvell mentioned that fusayama artificial saliva solution provides only theinorganic components, that is, NaCl 15.33, K-5.37, Ca 540, Po_4_, 4.23, Na_2_5-15.34, P_2_0_7_ 0.01, and Cl 23.02 (concentration of components are in mmoL/L) and does not permit the simulation of the effects of organic components, however, this electrolyte has a response close to natural saliva [25]. The actual conditions connected with the chemical and physical nature of the corrosive milieu are very complex, or even difficult to simulate the composition environment of oral milieu [14].

A study by Geis-Gerstorfer et al. [16] mentioned that, Ni-cr casting alloys do corrode and show average substance loss, varying between 0.540 and 3.26/mg/cm^2^after 35 days [7]; contrary to it, it is reported that there is good corrosion resistance for Ni-cr casting alloys in the oral cavity [9], but, in an in vitro study conducted by Chen et al. [19], mentioned that the Ni-cr casting alloys presented high resistance to corrosion. Thus, it can be mentioned that corrosion resistance is inversely proportional to corrosion rate; the more the value of corrosion rate is the least will be its corrosion resistance [26].

## 5. Conclusion

Within the limitations of this study, in correlation with literature, it can be concluded that Dentaurum's Remanium CS and Bego's Wiron 99 showed satisfactory corrosion behavior, with the exception of CB Soft of Sankin which depicted higher corrosion rate and least resistance to corrosion.

In the future development of alloys, an effort should be made to gain a better understanding of the interactions between the surface of the metal and its environment; a particular interest should be given to those between the physical and chemical state of alloy surface and its corrosion behavior.

## Figures and Tables

**Table 1:** Details of the metal alloys used.

Base metal alloy	Manufacturing company	Product name	Composition
Nickel chromium	Dentaurum	Remanium CS	Ni-61 wt%
			Cr-26 wt%
			Mo-11 wt%
			Si-1.5
			Fe
			Ce
			A1
			Co < 1

Nickel chromium	Bego	Wiron 99	Ni-65
			Cr-22.5
			Mo-9.5
			No:1
			Si −1
			Fe-0.5
			Ce-0.5
			C:0.02

Nickel chromium	Dentsply Sankin	CB Soft	Ni-72.8
			Cr-4.9
			Cu-12.3
			Other −10%

**Table 2:** Composition of fusayama artificial saliva solution.

0.96 grams (gms)	Kcl Potassium chloride
0.674 gms	Nacl sodium chloride
0.0405 gms	Mgcl_2_·6H_2_O magnesium chloride
0.117 gms	Cacl_2_·2H_2_O calcium chloride
0.091 gms	K_2_HPO_4_ potassium di hydrogen phosphate
0.11 gms	methyl parahydroxybenzoate
8.0 gm	70% sorbitol

**Table 3:** Mean values for corrosion rate (MPY).

Alloys	10th day	20th day	30th day
Dentaurum Remanium (CS)	17.0130506	47.1277852	16.61719
Bego (Wiron 99)	51.726802	44.1613956	82.0454818
Sankin (CB Soft)	26.8303936	61.8402746	293.692578

**Table 4:** Mean values for corrosion potential *E*_*corr*_ (mV).

	10th day	20th day	30th day
Dentaurum Remanium (CS)	431.18	441.78	492.58
Bego (Wiron 99)	470.86	493.28	744.08
Sankin (CB Soft)	567.58	587.32	740.58

**Table 5:** Mean values of corrosion current density *I*_*corr*_ (A).

	10th day	20th day	30th day
Dentaurum Remanium (CS)	3.51	3.736	3.092
Bego (Wiron 99)	4.95	3.818	4.622
Sankin (CB Soft)	5.998	6.554	3.376

**Table 6:** Mean values for corrosion resistance (K cm^−2^).

	10th day	20th day	30th day
Dentaurum Remanium (CS)	20.38	14.34	18.18
Bego (Wiron 99)	21.98	12.33	16.77
Sankin (CB Soft)	15.95	15.95	12.12
